# Hfq Is a Global Regulator That Controls the Pathogenicity of *Staphylococcus aureus*

**DOI:** 10.1371/journal.pone.0013069

**Published:** 2010-09-29

**Authors:** Yu Liu, Na Wu, Jie Dong, Yaping Gao, Xin Zhang, Chunhua Mu, Ningsheng Shao, Guang Yang

**Affiliations:** Beijing Institute of Basic Medical Sciences, Beijing, People's Republic of China; Research Institute for Children and the Louisiana State University Health Sciences Center, United States of America

## Abstract

The Hfq protein is reported to be an RNA chaperone, which is involved in the stress response and the virulence of several pathogens. In *E. coli*, Hfq can mediate the interaction between some sRNAs and their target mRNAs. But it is controversial whether Hfq plays an important role in *S. aureus*. In this study, we found that the deletion of *hfq* gene in *S. aureus* 8325-4 can increase the surface carotenoid pigments. The *hfq* mutant was more resistant to oxidative stress but the pathogenicity of the mutant was reduced. We reveal that the Hfq protein can be detected only in some *S. aureus* strains. Using microarray and qRT-PCR, we identified 116 genes in the *hfq* mutant which had differential expression from the wild type, most of which are related to the phenotype and virulence of *S. aureus*. Among the 116 genes, 49 mRNAs can specifically bind Hfq protein, which indicates that Hfq also acts as an RNA binding protein in *S. aureus*. Our data suggest that Hfq protein of *S. aureus* is a multifunctional regulator involved in stress and virulence.

## Introduction

Hfq protein was originally identified in *E. coli* as a host factor for the replication of the Qβ phage RNA [1] and is considered to be a multifunctional regulator of a variety of targets in bacteria [2]. Hfq regulates post-transcriptional gene expression in *E. coli*, specifically mRNA translation, stability or polyadenylation [3], [4].

Recently most studies have focused on the RNA chaperone function of Hfq in mediating the interaction between sRNA(small RNA) and mRNA [5]. In *E. coli*, some known sRNAs can interact with Hfq [6]. In *Salmonella*, Hfq associates with almost half of the co-immunoprecipitated sRNAs [7]. It is reported that Hfq can influence the stability of several sRNAs [8]–[10]. Besides acting as an RNA chaperone, *E. coli* Hfq has ATPase activity and can mildly affect transcription and translation *in vitro* [11], [12].

The inactivation of Hfq in *E. coli* can affect pleiotropic phenotypes, such as decreased growth rates, negative supercoiling of plasmids in stationary phase, increased carbon source oxidation, and increased sensitivity to ultraviolet light [13]. Hfq is also involved in stress response or virulence of a variety of pathogenic bacteria, such as *Neisseria meningitides, Salmonella*, *Vibrio cholerae* and *Pseudomonas aeruginosa* [7], [14]–[19].

*Staphylococcus aureus*, a major Gram-positive pathogen, can cause a broad range of diseases, from minor skin infections to life-threatening diseases, such as toxic shock syndrome and septicemia [20]. Many reports identify that RNAIII can act as antisense RNA and regulate the expression of several virulence genes, such as *spa* (Staphylococcal protein A, an important virulence factor of *S. aureus*) and *hla* (alpha-toxin gene, a major virulence factor of *S. aureus*) [21]. There is also an Hfq-like protein (8.9kD) in *S. aureus*, which can form homo-hexamer [22]. The structure of Hfq-binding RNA has been identified [3], [22]. Under *in vitro* condition, it is found that Hfq can specifically bind RNAIII and *spa* mRNA, which suggests that *S. aureus* Hfq can also act as an RNA chaperone [23]. However, Bohn *et al*. showed that Hfq did not play a crucial role in stress response, *spa* mRNA expression, or exoprotein expression in *S. aureus* RN6390, COL and Newman. They also tested ∼2000 phenotypes in the RN6390 *hfq* mutant by the Phenotype Microarray (PM) Technology [24], and found Hfq was not involved in the stress response, resistance to several chemical agents, or metabolic pathways [24]. It was also reported that *S. aureus* Hfq could not affect SA1000 expression, which is a fibrinogen-binding protein and identified as a target of RNAIII [25]. Geisinger *et al*. suggested that the weak transcription of *hfq* was possibly due to the loss of the gene's major promoter [26].

In this study, we found the carotenoid pigment increased in the *hfq* mutant of *S. aureus* 8325-4 (*Δhfq-8325*) and the mutant was less toxic during infection of MDBK cell and a mouse model of peritonitis than its parental strains. The results of the microarray studies showed that the expression of the 116 genes was altered in *Δhfq-8325*. We also found that 48 mRNAs of the 116 genes can specifically bind Hfq, which suggests that Hfq may be an RNA binding protein. We believe Hfq is a multifunctional regulator of gene expression and plays a major role in the infection of *S. aureus*. Our conclusion is contrary to the finding by Bohn *et al*. The difference of these findings may be due to differing expression patterns of the Hfq protein in different *S. aureus* strains.

## Results

### Deletion of Hfq in *S. aureus* 8325-4 increased the carotenoid pigments

**Figure 1:**
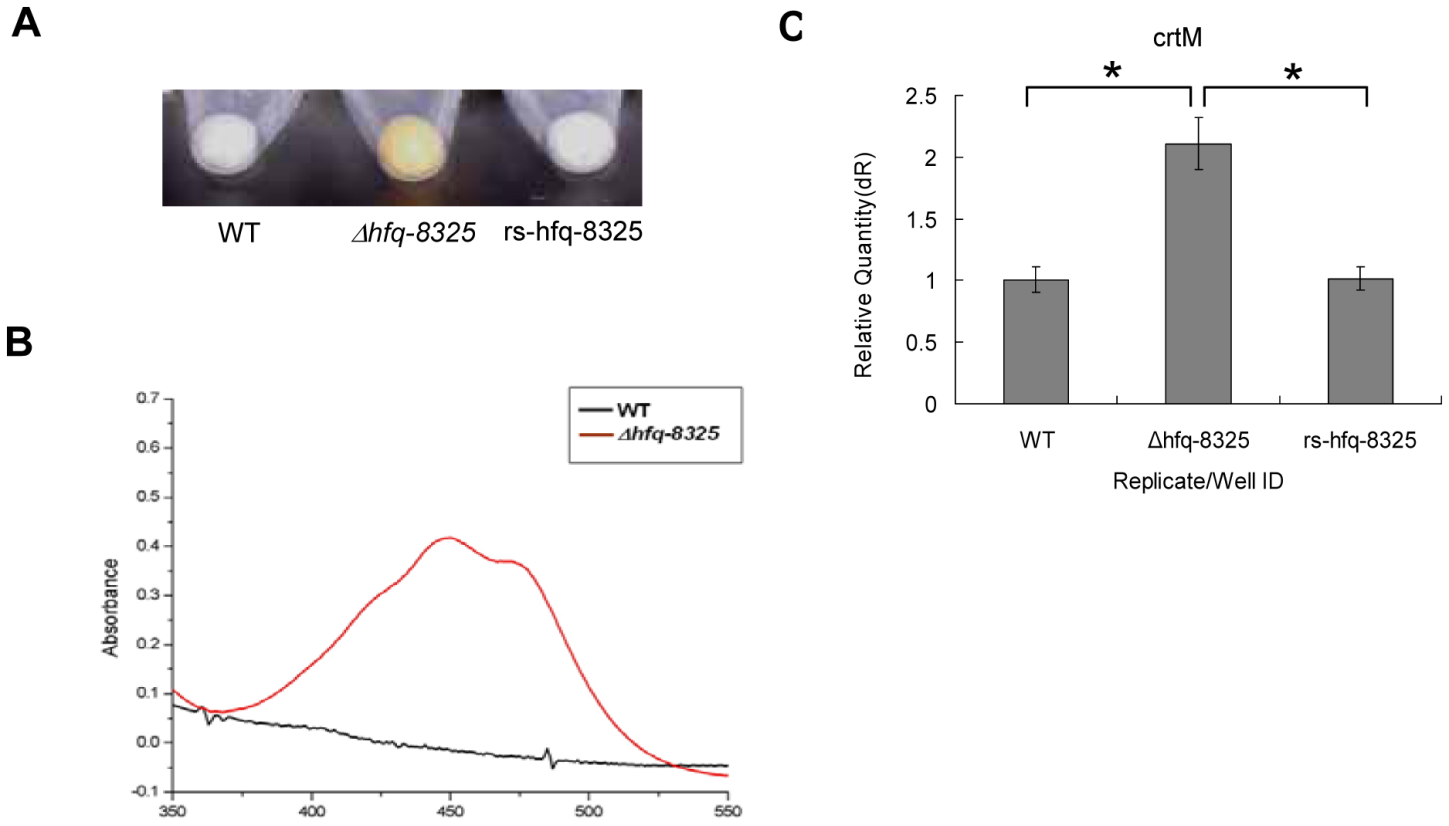
Effect of *hfq* deletion on pigmentation. *S. aureus* strains WT, *Δhfq-8325* and *rs-hfq-8325* were cultured for 12 h. The pigments of different strains were determined. The carotenoid pigment of *Δhfq-8325* was increased (**A**) and showed the characteristic triple peak spectral profile of carotenoid (**B**). However, the color of *rs-hfq-8325* was similar to the wild type. The expression level of *crtM* was detected by qRT-PCR. The results of qRT-PCR showed that the expression of *crtM* was increased in *Δhfq-8325* and recovered in *rs-hfq-8325* (**C**). The results shown were representative of three independent experiments (* P<0.01). WT, *S. aureus* 8325-4; *Δhfq-8325*, 8325-4 with an hfq::kan mutation; *rs-hfq-8325*, the restoration of Hfq activity in *Δhfq-8325*.

We constructed the *hfq* gene deletion mutant (*Δhfq-8325*) derived from *S. aureus* 8325-4. The result showed that *Δhfq-8325* displayed high intensities of yellow pigmentation compared to the wild type strain. After the restoration of Hfq activity in *Δhfq-8325*, the color of the restored strain (*rs-hfq-8325*) was recovered (**Figure 1A, 1B**).The intensity of the yellow carotenoid pigment of *S. aureus* is one of the classical criteria for identification of this species. The genes of *crtM* and *crtN,* which encode dehydrosqualene synthase and dehydrosqualene desaturase respectively, are essential for the pigment's synthesis of *S. aureus*. Both genes are located in the same operon [27]. We quantified the mRNA level of *crtM* in 8325-4, *Δhfq-8325* and *rs-hfq-8325* by qRT-PCR, and showed that the mRNA level of *crtM* increased in *Δhfq-*8325 (**Figure 1C**). This may explain why the carotenoid pigment of *Δhfq-8325* was increased.

**Figure 2:**
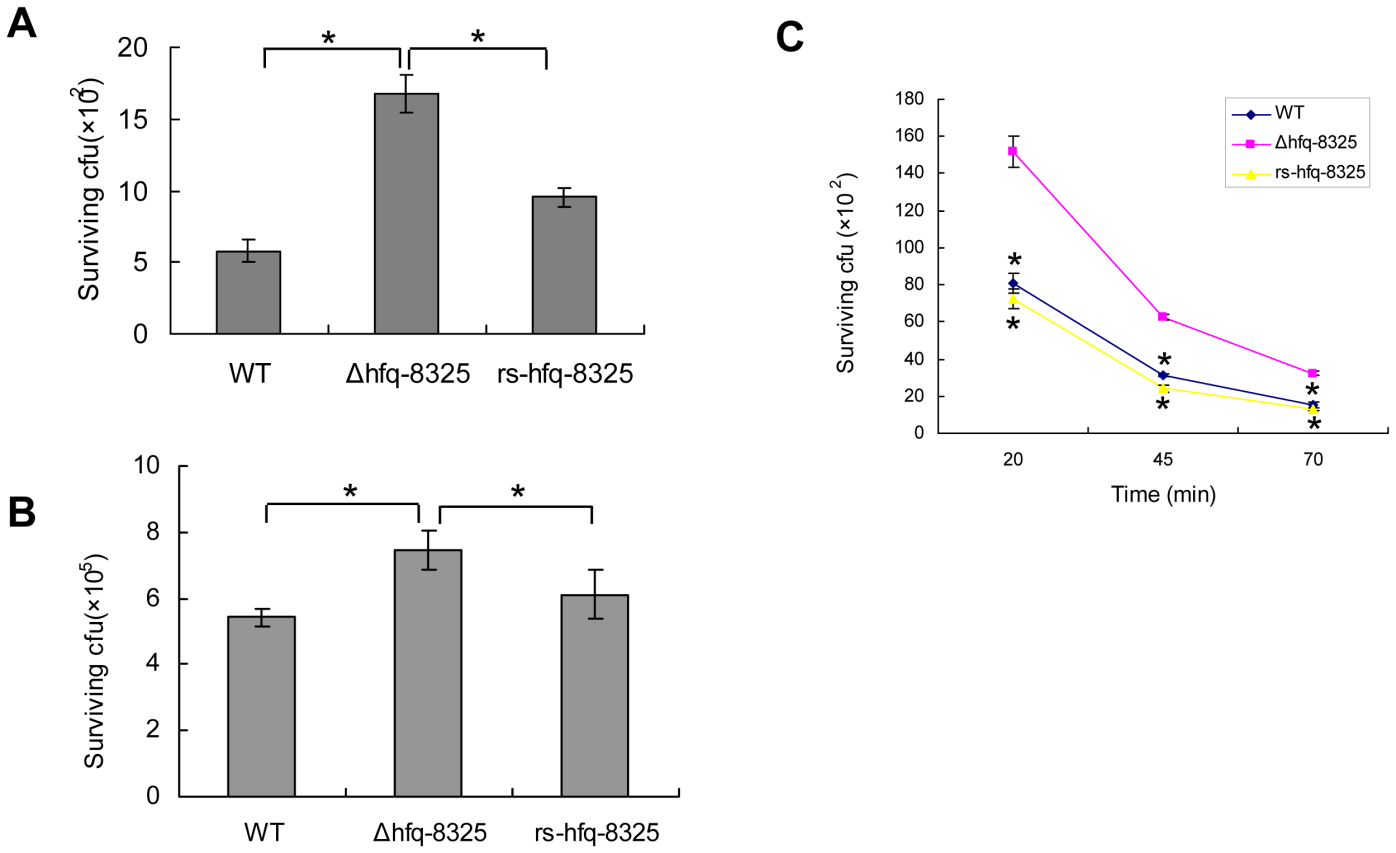
*Δhfq* -8325 shows enhanced resistance to oxidant and neutrophil killing. The oxidant susceptibility assays were performed to detect the susceptibility of *Δhfq-8325* to oxidative stress. *S. aureus* cells were incubated with oxidants or neutrophil. The survival number of bacteria was determined. The figures showed that *Δhfq-8325* conferred enhanced oxidant and neutrophil resistance compared with the wild type (*S. aureus* 8325-4). The results shown were representative of three independent experiments (* p<0.01). **A:** the singlet oxygen assay; **B:** the double oxygen assay; **C:** neutrophil survival assays. The results shown were representative of three independent experiments. WT, *S. aureus* 8325-4; *Δhfq-8325*, 8325-4 with an hfq::kan mutation; *rs-hfq-8325*, the restoration of Hfq activity in *Δhfq-8325*.

It was reported that the pigment acted as an antioxidant for *S. aureus*' resistance to neutrophil killing [28]. We tested the susceptibility of *Δhfq-8325* to oxidant, and showed that *Δhfq-8325* was indeed more resistant to oxidant than the wild type (**Figure 2A, 2B**). In addition, we found that the number of the mutant bacterium survived in neutrophils was significantly larger than that of the wild type (**Figure 2C**). We also found that *Δhfq-8325* didn't display other phenotypic variations including biofilm formation, cell growth, surface charge and hydrophobicity (data not shown).

**Figure 3:**
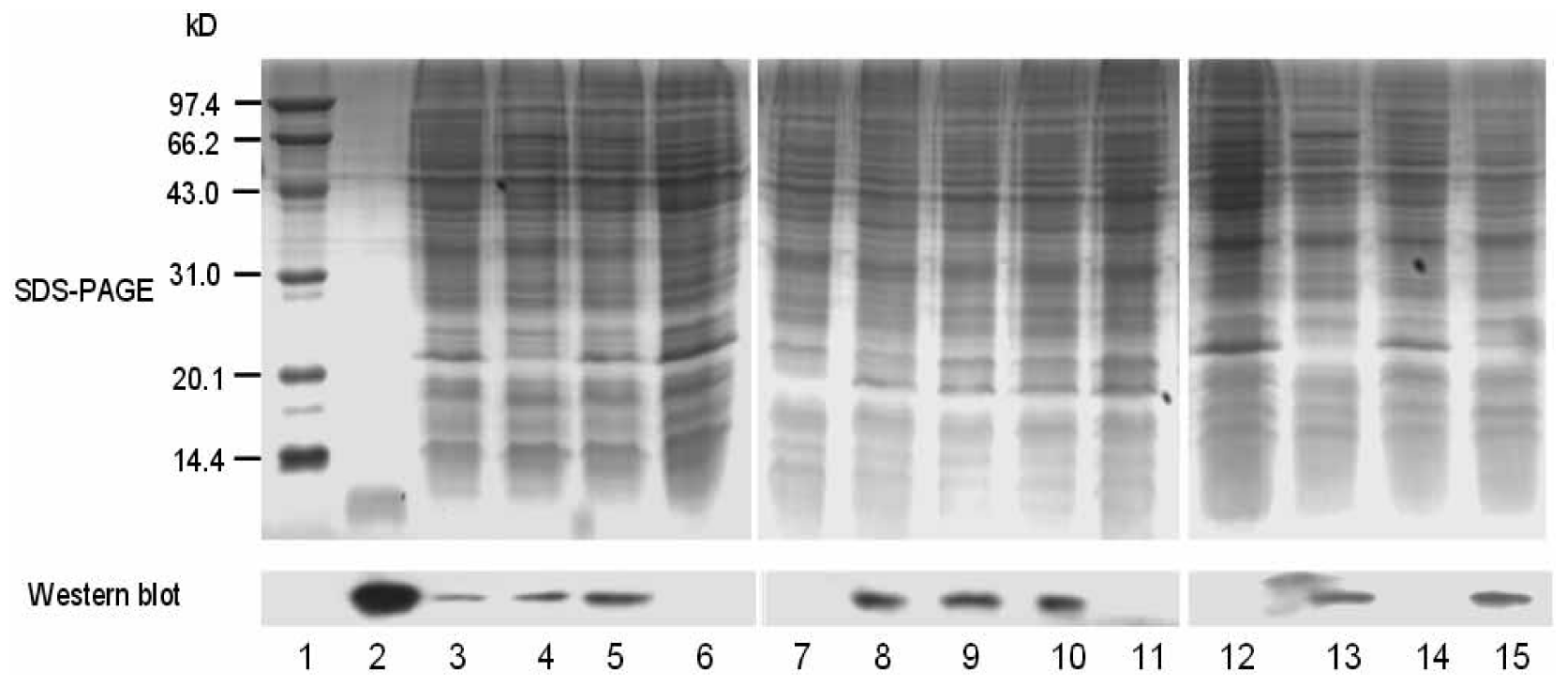
The detection of Hfq in the different *S. aureus* strains by Western blot. The total cellular proteins from the *S. aureus* cells at the same growth phase were extracted from different *S. aureus* strains, and the equal quantity of proteins was loaded to SDS-PAGE. The expression of Hfq was tested with the specific antibodies of Hfq by Western blot. The recombinant Hfq protein was used as a control. Lanes: 1, Molecular Weight Marker; 2, recombinant Hfq protein; 3, 040196; 4, 040188; 5, ATCC25923; 6, COL; 7, RN6390; 8, MRSA8963; 9, MRSA8972; 10, MRSA9004; 11, MRSA8973; 12, MRSA8979; 13, 8325-4; 14, *Δhfq-8325*; 15, *rs-hfq-8325*.

The bacterial strains used in the work of Bohn *et al.* were RN6390, COL and Newman, which were different from the ones in our work [24]. Although the mRNA of *hfq* could be detected in such strains by RT-PCR, we wondered whether this mRNA could be translated into the protein. So we extracted the same amount of total cellular proteins from 8325-4, RN6390 and other 11 strains, and detected the expression of Hfq by Western blot with the specific anti-Hfq antibodies prepared in our lab. The results revealed that Hfq protein could be found in 8325-4 and other 7 strains, but not in COL and RN6390 (**Figure 3**). The lack of Hfq may explain the non-detectable effect of Hfq in *S. aureus* COL and RN6390 in the work of Bohn *et al.* [24]. The sequences of all the genes encoding Hfq protein including their promoter regions in these strains are confirmed to be the same (data not shown). We also compared the transcription of *hfq* in 8325-4 and RN6390 by qRT-PCR using 16S rRNA as the control. No significant difference was observed (data not shown). This result suggested that there might be some post-transcription regulation of Hfq protein. Recently Becher *et al.* reported that mRNA of *hfq* (SACOL1324) could be detected in COL by DNA microarray, but the Hfq protein was undetectable [29]. This result is consistent with our observation. The different expression profiles of Hfq in *S. aureus* strains needs further investigation.

### *Δhfq-8325* was susceptive to the whole blood killing in spite of its increased golden pigments

**Figure 4:**
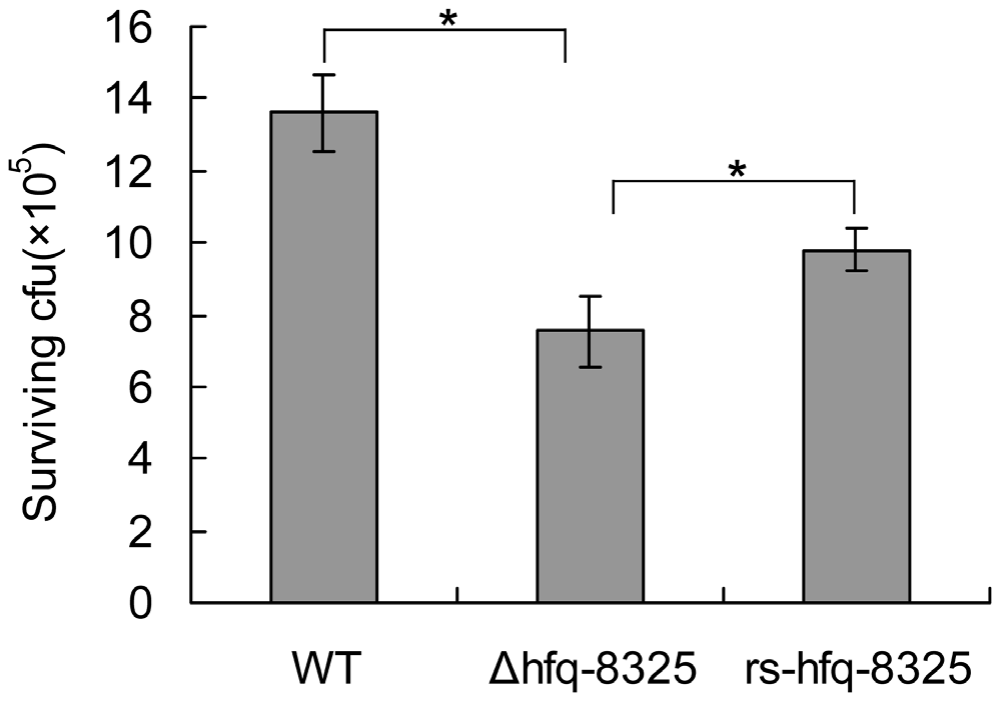
The survival number of different strains in whole blood. *S. aureus* cells were incubated with human whole blood. The survival bacterial number was detected. Compared with its parental strain, the *Δhfq-8325* strain was more sensitive to whole blood killing. The results shown were representative of three independent experiments (* p<0.01). WT, *S. aureus* 8325-4; *Δhfq-8325*, 8325-4 with an hfq::kan mutation; *rs-hfq-8325*, the restoration of Hfq activity in *Δhfq-8325*.

The work of Liu *et al*. showed that the number of *ΔcrtM-8325* survived in the whole blood decreased compared with WT because of reduced pigment synthesis [28]. We performed a similar experiment to verify whether *Δhfq-8325* was more resistant to the whole blood than wild type. It was surprising that the number of surviving *Δhfq-8325* cells was significantly less than the wild type (**Figure 4**). These results suggest that some other regulators in *Δhfq-8325* might be more important than the pigments for *S. aureus* survival in the whole blood.

### The deletion of *hfq* decreased the toxicity of *S. aureus*

**Figure 5:**
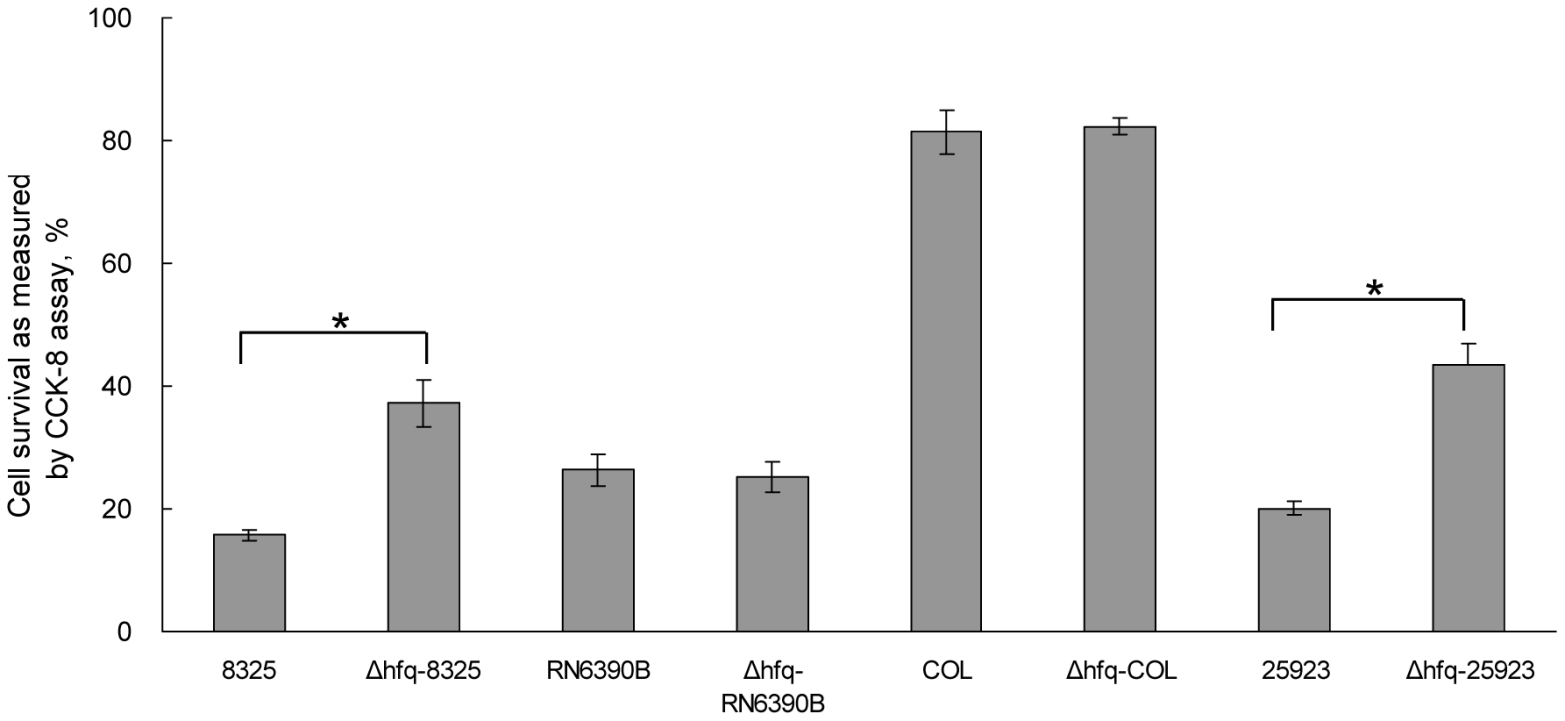
Detection of the percentage of survival cell using CCK8 assay. The supernatants from different strains were collected by centrifugation and incubated with MDBK cells for 12 h at 37°C. Cell survival was determined by CCK8 assay. The supernatant of *Δhfq-8325* was less cytotoxic than that of wild type (* p<0.01).

**Figure 6:**
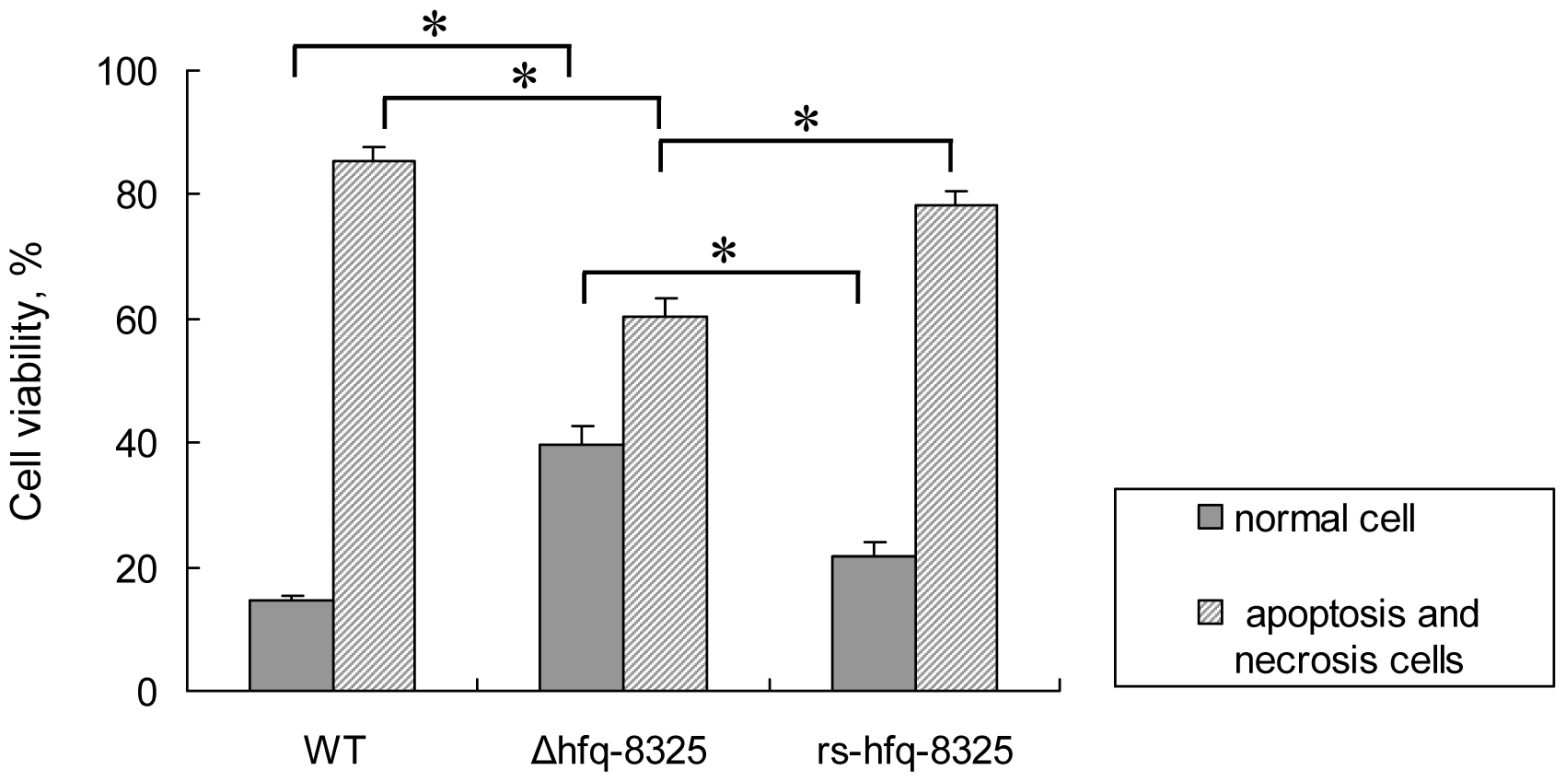
Flow cytometric analysis of the percentage of apoptosis and necrosis of cells. The apoptosis and necrosis of MDBK cells treated with the supernatant of *S. aureus* was detected by flow cytometry. The figure shows the summary data of the percentage of normal, apoptosis and necrosis of MDBK cells after the challenge of supernanant of different strains. The percentage of apoptosis and necrosis induced by the supernatant of *Δhfq-8325* was lower than wild type. The final data represented the mean ± SD for three independent experiments (* p<0.01).

**Figure 7:**
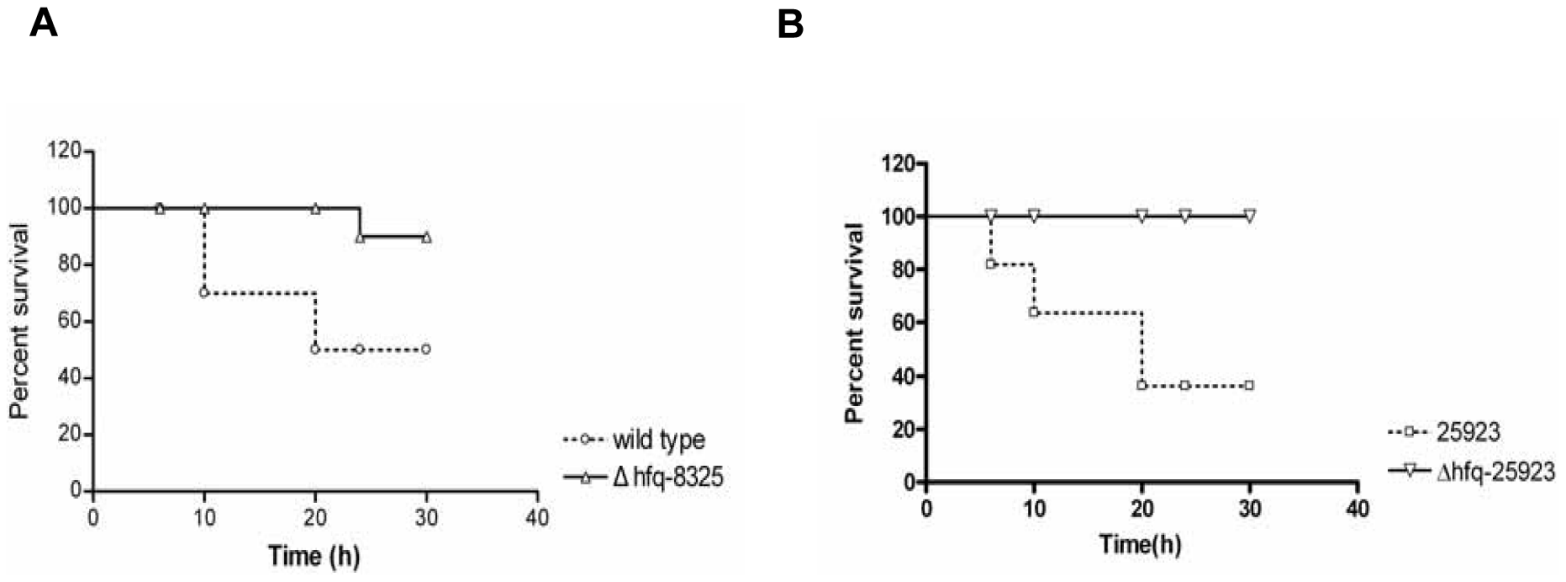
The pathogenicity of the *hfq* deletion mutant was decreased in the animal model. The pathogenicity of the *hfq* deletion mutants was assessed in a murine peritonitis model. *S. aureus* cells were injected intraperitoneally into the mice (10 mice per group). Survival of mice was recorded at the different time points. The result showed that the survival number of the *hfq* deletion mutants group was larger than that of the wild type group at each time point.

It is well known that the secreted toxins are important for infection caused by *S. aureus*. We compared the cytotoxicity of the *hfq* mutant with that of the wild type. After incubation with the supernatants from different strains, the survival cell numbers were quantified by CCK8 assay. We found that the toxicity of the supernatant of *Δhfq-8325* significantly decreased compared to its parental stain (**Figure 5**). However, the cytotoxicity of the supernatant of RN6390 and COL did not alter after the *hfq* gene deletion (**Figure 5**). In order to investigate if Hfq protein could influence the toxicity in other strains besides 8325-4, we constructed an *hfq*-deletion mutant from ATCC25923, which was an Hfq positive strain (**Figure 3**). Similarly, the survival percentage of cells after treatment of the supernatant of ATCC25923 was lower than that of the *hfq* mutant (**Figure 5**). The apoptosis and necrosis of MDBK cells induced by the supernatant was tested using flow cytometry. We found that the percentage of apoptosis and necrosis induced by the supernatant of *Δhfq-8325* was significantly lower than that of the wild type (**Figure 6**). In our further study, the pathogenicity of *Δhfq-8325* was assessed in a murine peritonitis model, with an equal number of bacterium from the wild type and *Δhfq-8325* injected intraperitoneally into the mice. The result showed that at the different time points (6 h, 10 h, 20 h, 24 h and 30 h), the survival number of the *Δhfq-8325* group was larger than that of the wild type group(**Figure 7A**), which was consistent with the results of the whole blood survival assay and the cytotoxicity assay. The pathogenicity of *Δhfq*-25923 was also assessed in the same murine peritonitis model, and the result showed that the survival number of the *Δhfq*-25923 group was larger than that of ATCC25923 group (**Figure 7B**).

### Identification of the changed genes in *?hfq-8325* using microarray assay

**Figure 8:**
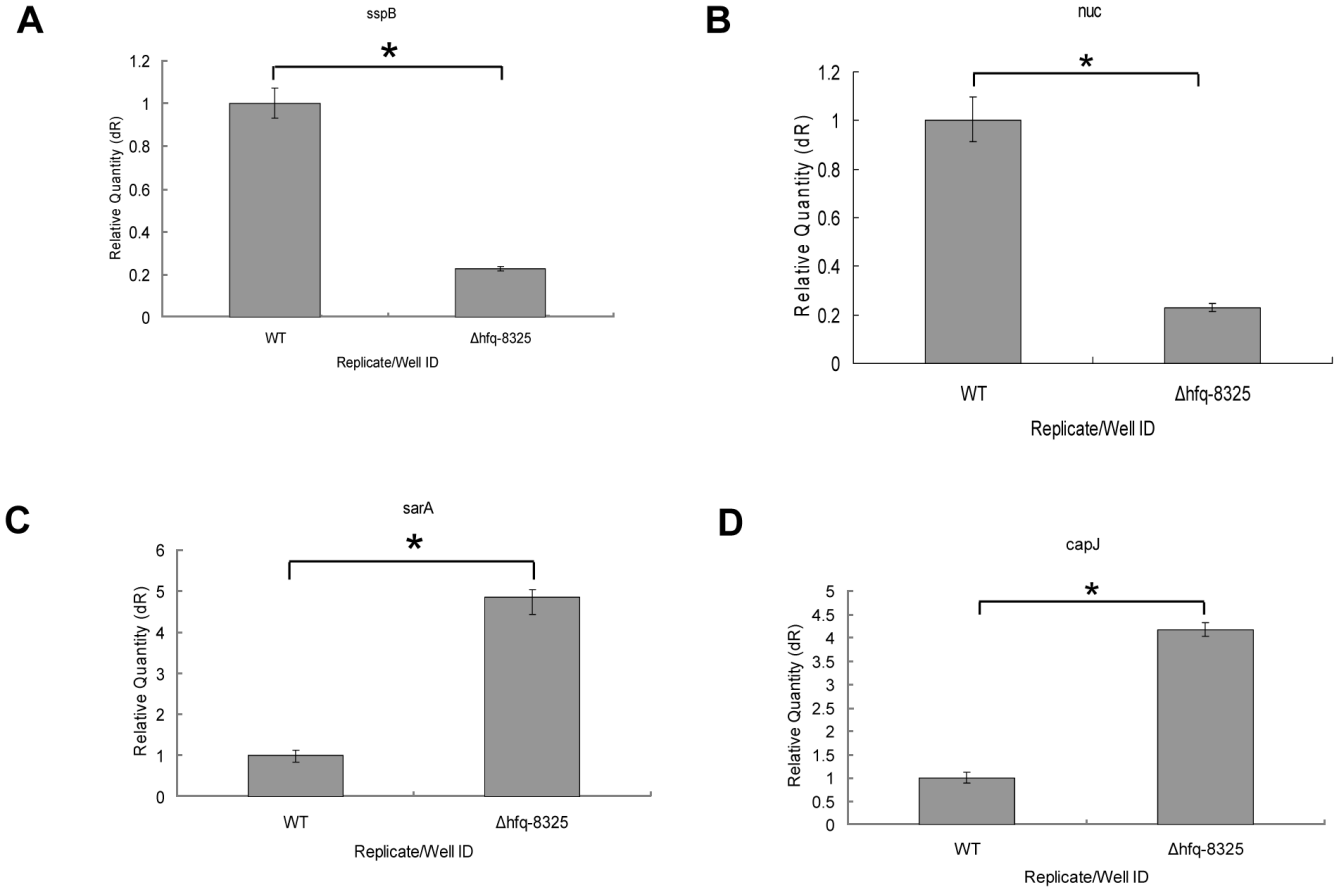
qRT-PCR quantification of the expression of *sspB, nuc, sarA* and *capJ*. The expression level of *sspB, nuc, sarA* **and** *capJ* was detected by qPCR. The results of qRT-PCR were accordant to the microarray data. The results shown were representative of three independent experiments (* P<0.01).

The phenotype and pathogenicity of *Δhfq-8325* were different from that of the wild type, so we analyzed the global change of gene expression in *Δhfq-8325* by microarray. The results revealed that the expression levels of 116 genes were altered in *Δhfq-8325* (fold change≥1.5), most of which were involved in the stress response and pathogenicity of *S. aureus*. Among the 116 genes, 33 had declined expression level (Table S1), while 83 had elevated expression level, compared to the wild type (Table S2). It was found that the level of *crtM* in *Δhfq-8325* was increased, which was consistent with the result of qRT-PCR. The expression of some virulence genes (such as serine protease, cysteine protease and staphylococcal nuclease) and global regulators (such as *sarA*) was changed after *hfq* deletion, which could affect the pathogenicity of *Δhfq-8325*. In order to validate the microarray data, the level of four genes (two up-regulated genes and two down-regulated in *Δhfq-8325*) was tested. The results of qRT-PCR were consistent with the microarray data (**Figure 8**).

Although it remained controversial whether Hfq plays an important role in *S. aureus*, it has been confirmed that Hfq protein could specifically bind RNAIII [23]. We obtained the RNAs that could potentially bind Hfq protein by immunoprecipitation *in vitro* and these RNAs were further analyzed by microarray. We identified about 300 mRNAs which were enriched in the pool of Hfq IP, including RNAIII (Table S3). Then we compared these RNAs with the 116 genes identified in *Δhfq-8325* (Table S1, S2), and we found that 49 of the 116 genes in *Δhfq-8325* were enriched in the pool of RNAs that bind Hfq protein. It is likely these genes may be regulated by Hfq.

## Discussion

Hfq was discovered as an *E. coli* host factor required for replication of RNA phage Qβ [1]. Hfq protein belongs to the large family of Sm and Sm like proteins. The sequence alignment shows that *E. coli* Hfq protein and *S. aureus* Hfq have the conserved Sm1 sequence motif, and the resolved structures show both of them can form homo-hexameric stuctures [3]. More recent studies show that Hfq protein can act as an RNA chaperone to mediate RNA-RNA interaction in *E. coli* [10]. But it is controversial whether Hfq plays an important role in *S. aureus*. Many reports reveal that *S. aureus* RNAIII acts as an antisense RNA to regulate its target genes expression [23], [25], [26], and it is a potential target of *S. aureus* Hfq *in vivo* [23]. However, Boisset *et al.* found that Hfq protein exerted no major effect *in vivo* on *SA1000*, *SA2353*, *rot* mRNA levels or RNAIII stability in *S. aureus* RN6390 [25]. Furthermore Bohn *et al.* reported that the deletion of Hfq had no noticeable effect on virulence gene expression nor was this protein highly expressed in *S. aureus* RN6390 [24].

In our work, we found that the deletion of Hfq can increase the pigments synthesis in 8325-4, while the color of the bacteria was recovered after the *hfq* gene was restored in *Δhfq-8325*. These results suggest that Hfq can influence the phenotype of *S. aureus*. The reason why our conclusion is different from that of Bohn *et al.* may be due to the different strains used in the experiments. Although *S. aureus* 8325-4 and RN6390 both originate from *S. aureus* 8325, the RN6390 has been generated by three rounds of transduction from 8325-4, and they have been validated to be different strains [30]–[32]. We also found that the Hfq protein can be detected in 8325-4 and other strains, but not in RN6390. The lack of Hfq expression may explain that there were no detectable changes induced by inactivation of Hfq in some strains. The reason why Hfq can not be detected in some strains is not clear and is now being investigated in our lab.

Although the pigments increased in *Δhfq-8325*, we found the survival number of *Δhfq-8325* in the whole blood decreased significantly and the *Δhfq-8325* was less toxic to mammalian cells and mice. These results suggest that the pigments are just one factor of *S. aureus* pathogenicity and there may be some other more crucial factors regulated by Hfq. We then compared the gene expression profile of *Δhfq-8325* with that of wild type. The transcription analysis showed that the expression of many genes was altered. Several serine protease and cysteine proteases (such as *sspA*, *sspB* and *splC*) were down-regulated in *Δhfq-8325* and identified as the important factors for *S. aureus* infection. A transposon mutant of the ssp operon had an attenuated virulence in three separate animal models (mouse abscess, bacteremia and wound infection) [33]. This may explain why the pathogenesis of *Δhfq-8325* was decreased in the animal model.

Some recent researches have suggested that the studies defining the regulatory roles of *sarA* and *agr* using RN6390 are not always representative of the events that occur in clinical isolates of *S. aureus* [34], [35]. So we constructed an *hfq* deletion mutant from ATCC25923, which is a clinical isolate, to investigate the biological function of Hfq. Our results showed that the cytotoxiticy and pathogenesis of the *Δhfq-25923* was significantly decreased as compared to its parental strain which suggests that Hfq can influence the toxicity of lab strains and clinical isolates.

The RNA chaperone activity of *S. aureus* Hfq has not been confirmed [23]–[25]. Although Hfq protein of *E. coli* has a longer C terminal-tail than that of *S. aureus*, the tail appears to be dispensable in the RNA binding function of Hfq [3]. Here we obtained the RNAs that bind Hfq in *S. aureus* 8325-4 by immunoprecipitation. The binding RNAs were then identified using microarray technology. We found that about 40% of the mRNAs of the changed genes in *Δhfq-8325* could specifically bind Hfq protein. The results suggest that the Hfq protein in *S. aureus* can also act as an RNA binding protein such as in *E. coli*. We are investigating the mechanism how these genes can be regulated by Hfq directly or indirectly.

## Materials and Methods

### Ethics Statement

All animal experimental protocols of the study are in accordance with the national guidelines for the use of animals in scientific research. It's also approved by Animal Care and Use Committee of Beijing Institute of Basic Medical Sciences, with the approval number BMS-081210.

### Bacterial strains and growth conditions

The strains used in this study are listed in Table 1. Strains were grown in 5 ml of brain heart infusion (BHI, BD) or Luria-Bertani (LB) medium at 37°C for 12 h with shaking at 200 rpm in a 25-ml test tube. Cells from 1 ml of pre-culture were transferred to 100 ml of BHI or LB medium in a 500-ml flask and incubated at 37°C on a rotary shaker at 200 rpm. *S. aureus* strains were routinely grown in BHI and *E. coli* strains were grown in LB medium either with no antibiotics, or with 20 µg/ml erythromycin, 100 µg/ml ampicillin and 50 µg/ml kanamycin.

**Table 1:** Bacterial strains and plasmids.

Strain or plasmid	Comments	Source or reference
**Strain**		
***S. aureus***		
*8325-4*	Wild-type, rsbU^-^	[30]
*Δhfq-8325*	8325-4 with an hfq::kan mutation	This study
*rs-hfq-8325*	*Δhfq-8325* restoring Hfq activity	This study
*ATCC25923*	clinical isolate	[42]
*Δhfq-25923*	ATCC25923 with an hfq::kan mutation	This study
*COL*	Highly methicillin resistant clinical isolate	Dr. William M. Shafer
*Δhfq- COL*	COL with an hfq::kan mutation	This study
*RN6390*	Laboratory virulent strain derived from 8325	[31]
*Δhfq-RN6390*	RN6390 with an hfq::kan mutation	This study
*RN4220*	Restriction-negative strain, 8325 derivative	[43]
*MRSA 8963*	Methicillin resistant clinical isolate	professor Jingui Cao
*MRSA 8972*	Methicillin resistant clinical isolate	professor Jingui Cao
*MRSA 8973*	Methicillin resistant clinical isolate	professor Jingui Cao
*MRSA 8979*	Methicillin resistant clinical isolate	professor Jingui Cao
*MRSA 9004*	Methicillin resistant clinical isolate	professor Jingui Cao
*040188*	Laboratory strain	stored in our lab
*040196*	Laboratory strain	stored in our lab
***E. coli***		
*DH5α*	A host strain for cloning	Transgene
*BL21(DE3)*	A host strain for protein expression	Transgene
**Plasmids**		
pMD19T	*E.coli* cloning vector, amp^R^	TaKaRa
pMD20T	*E.coli* cloning vector, amp^R^	TaKaRa
pET28 (a)	*E.coli* expression vector, kan^R^	Novagen
pAUL-A	Temperature-sensitive *S. aureus* suicide vector; Em^R^	[44]
pAUL-A-Δhfq	pAUL-A containing whole *hfq* gene	This study
pMAD	Temperature-sensitive S. aureus suicide vector; Em^R^	[45]
pMAD-rs-hfq	pMAD containing whole *hfq* gene for restoring Hfq activity	This study

### Expression and purification of Hfq protein

The *hfq* gene was amplified from the genome of *S. aureus* 8325-4 with the primers (hfq *Nco*I and hfq *Xho*I) in Table 2. The PCR product was cloned into the expression vector pET28(a) as an *Nco*I-*Xho*I fragment, generating pET28(a)-hfq, which was subsequently transformed into *E. coli* strain BL21 (DE3). Expression of the recombinant Hfq protein was induced by adding 0.1 mM isopropyl β-D-thiogalactopyranoside (IPTG) to a growing culture at OD_600_ = 0.4. Purification of recombinant protein on Q Sepharose Fast Flow and SP Sepharose Fast Flow columns (Amersham Biosciences) was carried out according to the manufacturer's instructions.

**Table 2:** Sequences of forward and reverse primers used in this study.

Primer/sequence	Oligonucleotide sequence (5′ to 3′)
hfq NcoI	CATGCCATGG GCATGATTGCAAACGAAAACAT
hfq XhoI	CCGCTCGAGTTATTCTTCACTTTCAGTAGATG
16S F	GCCTAATACATGCAAGT
16S R	CATGTTATCCGGCATTAG
Up-hfq F-EcoRI	CATCCGGAATTCCCAATCATAGCAGGTGGAAC
Up- hfq R-KpnI	TCACTTGGTACCCTGTCGGACTCCTTTTACT
Down- hfq F-KpnI	CGACAGGGTACCAAGTGAAGAATAAGTTGTAA
Down- hfq R-SalI	ACACGCGTCGACCAAATAAAGG TGTTACTTAC
rs-hfqF	TCTAGATAGTCGACTATTCACCCTAACAA
rs-hfqR	GATATGCATGAATTCATGGAGACAATTTAT
crtM RT primerF	GACTTGGTGAATCGTTGC
crtM RT primerR	CTATGATTGGTGATGCTTC
sspB RT primerF	AAACGAAGCGATACAAGA
sspB RT primerR	CATTTGATTAGGGAATGTTG
nuc RT primerF	GTAGCCATCATTATTGTAG
nuc RT primerR	CCATAGCGATCTTGTTTT
sarA RT primerF	CTCAAGAAGATTACTTCGAT
sarA RT primerR	GCTTCAGTGATTCGTTTA
capJ RT primerF	ATATCTACAAGGTGGAAC
capJ RT primerR	CCCTAATAAGCCAAATGA

### Production of polyclonal antibodies against Hfq

Female New Zealand White rabbits were first immunized by subcutaneous injection with 1 mg of Hfq protein in Phosphate Buffered Saline (PBS) mixed with complete Freund's adjuvant in a total volume of 1 ml. And the subsequent booster injections, i.e., 1 mg of Hfq in PBS emulsified in incomplete Freund's adjuvant, were administered at 3 and 6 weeks after the primary immunization. At the eighth week, the sera were collected and the antibody titers were determined by ELISA.

### Construction of *hfq* deletion mutant

The mutant was constructed using the method described previously [36] with some modifications. In order to create a deletion mutant of *hfq* in the chromosome of 8325-4, two regions of DNA flanking the *hfq* gene were amplified by PCR using the primers (Up-hfq F-EcoRI and Up-hfq R-KpnI; Down-hfq F-KpnI and Down-hfq R-SalI) with restriction sites listed in Table 2. The upstream fragment (659 bp) was digested with *Eco*RI and *Kpn*I, and the downstream fragment (680 bp) was digested with *Kpn*I and *Sal*I. The two fragments were cloned together into pMD19T digested with *Eco*RI and *Sal*I. The resulting construct was digested with *Kpn*I, and then a 1.6-kb kanamycin cassette which was amplified from the plasmid of pTZ-TRAP::kan provided by Dr. Balaban N was inserted. The resulting plasmid was digested with *Eco*RI and *Sal*I, and a fragment harboring kanamycin resistance between the upstream and downstream fragments was ligated into pAUL-A digested with *Eco*RI and *Sal*I to create plasmid pAUL-A-Δhfq. pAUL-A has a temperature-sensitive origin of replication that is active in *S. aureus* at 30°C but not at 42°C. The recombinant plasmid initially isolated from *E. coli*, was introduced into *S. aureus* RN4220 by electroporation and colonies resistant to kanamycin and erythromycin were selected after growth at 30°C. The resistant clones were subjected to a temperature shift to 42°C to select the plasmid integration into the chromosome. Bacteria resistant to kanamycin but sensitive to erythromycin were selected. The mutation was confirmed by PCR, and followed by transduction into strains 8325-4, RN6390, COL and ATCC25923 with phageΦ11 to create the mutant strains from which the coding region of *hfq* (234 bp) was deleted (Table 1).

### Restoring Hfq Activity

Primers rs-hfqF and rs-hfqR (listed in Table 2) were designed to PCR-amplify a 1406 bp fragment encompassing the gene encoding for Hfq, the promoter region and termination site with *Sal*I/*Eco*RI sites. *S. aureus* 8325-4 chromosomal DNA was used as a template. The PCR product (“whole *hfq*”) was digested and cloned into the *Sal*I/*Eco*RI digested pMAD, which could replicate in *E. coli* at 37°C and in *S. aureus* at 30°C and could be used as a suicide vector when grown at 42°C in *S. aureus*. The resulting plasmid (pMAD-rs-hfq) was used to transform into *E. coli* DH5α. Cells were selected on LB plates containing 100 µg/ml ampicillin. Plasmid was isolated from positive clones and used to transform *S. aureus* RN4220. The transformants were selected on tryptic soy agar plates containing 10 µg/ml erythromycin at 30°C. Then the plasmid was isolated from the positive clone (RN4220 containing pMAD-rs-hfq) and transformed to Δ*hfq-8325* by electroporation. The transformants were selected on tryptic soy agar plates containing 10 µg/ml erythromycin at 30°C for 48 h, and then transferred to 42°C for 12 h. The restoring colonies (*rs-hfq-8325*) were confirmed by PCR and RT-PCR analysis.

### Oxidant susceptibility assay

The assay to test susceptibility to oxidants was performed as previously described [37] with some modifications. For hydrogen peroxide susceptibility assay, *S. aureus* cells were grown to early exponential phase and harvested by centrifugation. Hydrogen peroxide (H_2_O_2_) was added at a 1.5% final concentration to the bacteria (2×10^9^/ml) and incubated at 37°C for 1 h. Then, 1,000 U/ml of catalase (Sigma-Aldrich) was added to quench residual H_2_O_2_. Dilutions were plated on tryptic soy agar plates for enumeration of surviving cells.

The assay to test susceptibility to singlet oxygen was performed as previously described [37] with some modifications as well. *S. aureus* strains were grown to early exponential phase and harvested by centrifugation. The bacteria (10^8^/ml) were incubated at 37°C in a 24-well culture plate. 200 µl of 0.1 µg/ml methylene blue was added to the wells and the plate was situated at exactly 10 cm from a 100-W light source. The viable cells were assessed after 1.5 h by plating dilutions on tryptic soy agar plates.

### Neutrophil intracellular survival assay

Neutrophils for intracellular survival assays were purified from healthy human volunteers as previously described [37]. Bacteria were washed twice in PBS, diluted to a concentration of 4.5×10^6^ CFU (colony forming unit) in 100 µl RPMI1640 with 10% fetal calf serum and mixed with 3×10^5^ neutrophils in the same media. The mixture was centrifuged at 700 g for 5 min, and then incubated at 37°C in a 5% CO_2_ incubator. After 10 min gentamicin was added to a final concentration of 400 µg/ml for killing the extracellular bacteria. Then, the contents of sample wells were withdrawn and centrifuged to pellet the neutrophils. Neutrophils were washed twice, and then lysed in 0.02% Triton X-100. CFU was calculated by serial dilution plated on Todd-Hewitt agar (THA; 1.5% agar in Todd-Hewitt broth) plate.

### Whole-blood killing assay (WBKA)

*S. aureus* strains were grown for 12 h and harvested by centrifugation (10,000 g, 1 min). Bacteria were washed twice in PBS, and then 1×10^4^ cells were diluted to the volume of 25 µl in PBS, and mixed with 75 µl freshly drawn mouse blood in heparinized tubes. The tubes were incubated at 37°C for 4 h with agitation, and then surviving cells were enumerated by plating the dilutions on THA plates.

### Cytotoxicity assay

The supernatant of *S. aureus* was collected as described [38], [39] with some modifications. Briefly, *S. aureus* cells were grown to stationary phase at 37°C. Growth culture was centrifuged at 6,000 g for 10 min at 4°C. The supernatant was collected and filtered through a 0.22 µm filter to remove residual cells.

Madin-Darby Bovine Kidney (MDBK, ATCC-CCL22) cells were resuspended at a concentration of 1×10^5^ cells/ml and added to a 96-well plate (100 µl/well) at 37°C in a 5% CO_2_ incubator for 6 h. The supernatant of *S. aureus* strains (5 µl/well) was added, with BHI broth as the control. The CCK8 assay was used to test the number of survival cells as per the protocol (Dojindo) after 12 h incubation. The cell viability percentage was calculated as: Viability percentage (%)  =  (Absorption value of supernatant of treatment group)/(Absorption value of supernatant of control group) ×100%.

For flow cytometric analysis, the MDBK cells were resuspended at a concentration of 1×10^6^ cells/ml and added to a 12-well plate (1 ml/well) at 37°C in a 5% CO_2_ incubator for 6 h. The cultivated cells were incubated with BHI or with supernatant (100 µl/well) of *S. aureus* strains for 1 h. Prior to harvesting, the cells were washed twice with PBS, trypsinized, and pelleted. Then cells were resuspended at a concentration of 1×10^6^ cells/ml in Binding Buffer (0.01 M HEPES/NaOH, pH 7.4, 14 mM NaCl, 0.25 mM CaCl_2_). 500 µl aliquots of cells were added into FACS tubes and mixed with 25 ng/ml fluorescein isothiocyanate–labeled annexin V and 10 mg/ml propidium iodide (PI) to incubation for 15 min at room temperature in the dark. Then the cells were analyzed immediately by flow cytometry.

### RNAs binding to Hfq were obtained by immunoprecipitation

The immunoprecipitation assays were prepared as described previously [23] with some modifications. The purified Hfq protein (100 µg) was incubated with 5 ml rabbit immune serum containing *S. aureus* Hfq-specific antibodies or rabbit normal serum at 37°C for 30 min. The captured antibodies were then bound to 1 ml of protein A sepharose CL-4B (Sigma-Aldrich) at 37°C for 30 min. After four washes with 2 ml washing buffer (50 mM Tris–HCl, pH 8.0, 150 mM NaCl, 0.5% Triton X-100), the total RNA of *S. aureus* was added and incubated at 37°C for 30 min. After another four washes with 2 ml washing buffer, the binding complexes were eluted from protein A sepharose with 1 ml of 0.1 M Glycine–HCl, pH 2.5, and neutralized with 100 ul of 1 M Tris–HCl, pH 8.0. Then the mixture was extracted by phenol, followed by RNA precipitation and the RNAs which were bound to Hfq were analyzed by microarray. Calculation of enrichment factor was performed as follows: enrichment factor  =  signal intensities of Hfq IP/signal intensities of control IP.

### Transcriptional microarray analysis

Gene expression profiles of the *Δhfq-8325* and wild type strains were analyzed by using Affymetrix *S. aureus* arrays as described previously [40] with some modifications as follows. The *S. aureus* array contains probe sets to over 3,300 *S. aureus* open reading frames (ORFs) and 4,800 intergenic region sequences based on the updated *S. aureus* genomic sequences of N315, Mu50 and COL [41]. Total bacterial RNA was extracted from *S. aureus* which were grown for 6 h with shaking at 37°C using Trizol (Invitrogen) as previously described [41]. DNase digestion of 80 µl of total RNA was performed with 10 U of RNase-free DNase I (Promega) and 10 µl of the 10× reaction buffers in a total reaction volume of 100 µl for 30 min at 37°C. The RNA (10 µg) was reversely transcribed to cDNA by using M-MLV reverse transcriptase (Promega) and random primers (Promega) for 1 h at 42°C. The fragmented cDNAs were then directly labeled with biotin by using a biotin-ddUTP kit (Affymetrix). Biotinylated cDNA was hybridized to the GeneChips. Microarrays were scanned using an Affymetrix GeneChip Scanner 3000 and image data were extracted using GeneChip Operating Software (GCOS) Version 1.4. Two independent labeling reactions and hybridizations were carried out for each RNA sample. This experiment was completed by Bioassay Laboratory of CapitalBio Corporation. Normalization and expression analysis were performed with DNA-chip analyzer (dChip). Invariant set normalization was used to normalize arrays at the probe level. Genes with variations (n-fold≥1.5) were collected. All data is MIAME compliant, and the raw data has been deposited in the database ArrayExpress (No. E-TABM-1005).

### Acute murine peritoneal infection model

Groups (n = 10) of 6- to 8-week-old, male Balb/c mice were injected intra-abdominally with 500 µl of different strains. The injected cell number of *Δhfq-8325* or *S. aureus* 8325-4 was 1×10^8^ CFU per mouse. And the injected cell number of *Δhfq-*25923 or ATCC25923 was 5×10^8^ CFU per mouse. The survival number of mice was recorded at the different time points (6 h, 10 h, 20 h, 24 h and 30 h) post challenge. Survival outcomes in the wild type or the *Δhfq-8325* groups were compared.

### Preparation of *S. aureus* total proteins and Western blot

The cell extract was prepared as described [41] with some modifications as follows. Cells were grown for 6 h with shaking at 37°C. Equal numbers of cells (1×10^10^ CFU) were collected and then resuspended in 1 ml PBS buffer containing lysostaphin (100 µg/ml, Sigma-Aldrich). After incubation for 30 min at 37°C, the mixture was centrifuged at 13,000 g for 10 min and the precipitate was resuspended in 1 ml PBS. The mixture was sonicated on ice three times for 30 s each and centrifuged at 13,000 g for 10 min. The supernatant was precipitated by adjusting filtered supernatants to 10% trichloroacetic acid (TCA) and incubated at 4°C for 4 h. After centrifugations at 12,000 g for 10 min, precipitated proteins were washed twice in ice-cold 96% ethanol, and air dried. The proteins were resolved in an appropriate volume of a solution containing 7 M urea and 2 M thiourea. The samples were then subjected to 15% SDS-PAGE and the proteins were blotted onto Hybond-ECL nitrocellulose membrane (Amersham Biosciences). The membrane was blocked in 5% non-fat dry milk at 37°C for 2 h, probed with 1∶500 diluted polyclonal rabbit anti-Hfq antibodies (prepared by ourselves) for 1 h at room temperature, and washed twice in PBS with 0.5% Tween 20 (PBST). Then the membrane was incubated in a 1∶5,000 solution of HRP-conjugated goat anti-rabbit secondary antibody (Jackson) at room temperature for 1 h. After further washing with PBST, the membrane was assayed by the enhanced chemiluminescence (ECL) Western blotting detection system (Pierce).

### Quantitative reverse transcription PCR (qRT-PCR)

For cDNA synthesis, 1 µg of total RNA was mixed with 500 ng of random hexamer (Promega). Samples were incubated at 65°C for 10 min with 5 µl of 5× first-strand buffer, 2 µl of 5 mM dNTP, 2 0U of RNasin (Takara), 1 µl of M-MLV reverse transcriptase (Promega) and distilled water to a total volume of 25 µl. The qPCR reaction mixture contained 12.5 µl of 2×SYBR green PCR mix (GenePharma), 0.3 µM of gene-specific forward and reverse primers, and 1 µl of template, made up to a final volume of 25 µl with distilled water. The primers are shown in Table 2. Cycling parameters were set as follows: initial activation step at 95°C for 10 min, denaturation at 94°C for 30 s, annealing at 58°C for 30 s, and extension at 72°C for 40 s. Melting curve analysis was performed at from 58°C to 95°C with stepwise fluorescence acquisition at every 1°C s^−1^. Melting curves observed for each gene were confirmed to correspond to the correct amplicon size by agarose gel electrophoresis of the PCR products. The levels of gene expression were calculated by relative quantification using 16S rRNA as the endogenous reference gene. All samples were amplified in triplicate and the data analysis was carried out using the MxPro qPCR system software (Stratagene).

### Statistical analysis

All quantitative data were analyzed using Student t-tests. P<0.05 was considered to be statistically significant.

## Supporting Information

Table S1 Genes down-regulated in *Δhfq-8325*.
(0.07 MB DOC)

Table S2 Genes up-regulated in *Δhfq-8325*.
(0.14 MB DOC)

Table S3 Genes which were enriched in the pool of Hfq IP compared to the negative control.
(0.43 MB DOC)
