# Supplementary material for: Hfq Is a Global Regulator That Controls the Pathogenicity of Staphylococcus aureus
Source: PLoS One. 2010 Sep 29;5(9):e13069. doi: 10.1371/journal.pone.0013069 (PMC2947504; doi:10.1371/journal.pone.0013069)
Supplement: Table S1 — Genes down-regulated in Δhfq-8325. (0.07 MB DOC) [file pone.0013069.s001.doc]

**Table S1. Genes down-regulated in *Δhfq-8325***

| group function | MU50 ORF | MU50 gene | MU50 gene product | Fold change(WT/H) | Binding to Hfq^a^ | Enrichment^b^ |
| --- | --- | --- | --- | --- | --- | --- |
| Virulence Factors and Regulators | SAV1047 | sspB | cysteine protease precursor | 3.8 | + | 2.5 |
|  | SAV1813 | splA | serine protease | 3.6 | + | 27.0 |
|  | SAV1048 | sspA | serine protease | 3.4 | + | 11.2 |
|  | SAV1811 | splC | serine protease | 3.3 | + | 21.0 |
|  | SAV1809 | splF | serine protease | 3.3 | + | 4.2 |
|  | SAV1938 |  | truncated map-w protein | 2.9 | - |  |
|  | SAV1046 | sspC | cysteine protease | 2.8 | + | 10.2 |
|  | SAV1812 | splB | serine protease | 2.6 | + | 3.3 |
|  | SAV0815 | nuc | staphylococcal nuclease | 1.9 | + | 2.0 |
|  | SAV2637 | aur | zinc  metalloproteinase aureolysin | 1.9 | - |  |
|  | SAV0812 |  | staphylocoagulase precursor | 1.8 | - |  |
|  | SAV2202 | hysA | hyaluronate lyase precursor | 1.7 | - |  |
|  | SAV0133 | sodM | superoxide dismutase | 1.7 | + | 8.8 |
|  | SAV2502 | fnbB | fibronectin-binding protein homolog | 1.6 | - |  |
|  | SAV0263 | lrgB | antiholin-like protein | 1.5 | + | 4.0 |
|  | SAV1248 | eprH | endopeptidase resistance gene | 1.5 | - |  |
|  | SAV0433 |  | exotoxin 15 | 1.5 | + | 2.4 |
|  | SAV0424 | set8 | exotoxin 8 | 1.5 | - |  |
|  | SAV0262 | lrgA | murein hydrolase regulator | 1.5 | - |  |
|  | SAV2366 | lctP | L-lactate  permease  lctP homolog | 1.5 | + | 2.4 |
| Metabolism | SAV0241 | lctE | L-lactate dehydrogenase | 2.0 | + | 5.8 |
|  | SAV1066 | purC | phosphoribosoylaminoimidazole-succinocarboxamide synthase | 2.4 | - |  |
|  | SAV1068 | purQ | phosphoribosylformylglycinamidine synthase I | 2.8 | - |  |
|  | SAV1067 | purS | phosphoribosylformylglycinamidine synthase | 2.9 | - |  |
|  | SAV1069 | purL | phosphoribosylformylglycinamidine synthetas | 2.9 | - |  |
|  | SAV1071 | purM | phosphoribosylaminoimidazole synthetase | 3.5 | - |  |
|  | SAV1074 | purD | Phosphoribosylamine  -glycine ligase | 3.6 | - |  |
|  | SAV1070 | purF | phosphoribosylpyrophosphate amidotransferase | 3.8 | - |  |
|  | SAV1072 | purN | phosphoribosylglycinamide formyltransferase | 3.9 | - |  |
| Protein Synthesis | SAV1073 | purH | bifunctional purine biosynthesis protein | 3.8 | + | 2.0 |
| hypothetical protein | SAV2205 |  | hypothetical protein | 2.0 | + | 8.5 |
|  | SAV1738 |  | hypothetical protein | 2.0 | + | 3.6 |
|  | SAV0766 |  | hypothetical protein | 2.2 | - |  |

a:+, binding to Hfq; ­-, not binding to Hfq.

b: Enrichment factor calculated by the signal intensities of Hfq IP over control IP.
