# Supplementary material for: Hfq Is a Global Regulator That Controls the Pathogenicity of Staphylococcus aureus
Source: PLoS One. 2010 Sep 29;5(9):e13069. doi: 10.1371/journal.pone.0013069 (PMC2947504; doi:10.1371/journal.pone.0013069)
Supplement: Table S2 — Genes up-regulated in Δhfq-8325. (0.14 MB DOC) [file pone.0013069.s002.doc]

**Table S2. Genes up-regulated in *Δhfq-8325***

| group function | MU50 ORF | MU50 gene | MU50 gene product | Fold change  (H/WT) | Binding to Hfq^a^ | Enrich  ment^b^ |
| --- | --- | --- | --- | --- | --- | --- |
| Virulence Factors and Regulators | SAV1816 | epiG | epidermin immunity protein F | 6.3 | - |  |
|  | SAV1174 |  | antibacterial  protein (phenol soluble modulin) | 3.7 | - |  |
|  | SAV0616 | sarA | staphylococcal accessory regulator A | 3.1 | + | 3.6 |
|  | SAV0811 | fnb | fibrinogen-binding protein | 2.9 | - |  |
|  | SAV0158 | capJ | capsular polysaccharide synthesis enzyme Cap5J | 2.8 | - |  |
|  | SAV0498 | spoVG | stage V sporulation protein G homolog | 2.6 | + | 6.8 |
|  | SAV2386 | sarZ | staphylococcal accessory protein Z | 2.4 | + | 9.3 |
|  | SAV0157 | capI | capsular polysaccharide synthesis enzyme Cap5I | 2.4 | - |  |
|  | SAV0159 | capK | capsular polysaccharide synthesis enzyme Cap5K | 2.2 | - |  |
|  | SAV1553 | sodA | superoxide dismutase | 2.2 | + | 9.7 |
|  | SAV0163 | capO | capsular polysaccharide synthesis enzyme Cap8O | 2.2 | - |  |
|  | SAV0513 | cysK | cysteine synthase | 2.1 | - |  |
|  | SAV0162 | capN | capsular polysaccharide synthesis enzyme Cap5N | 2.1 | - |  |
|  | SAV0160 | capL | capsular polysaccharide synthesis enzyme Cap5L | 2.1 | + | 2.1 |
|  | SAV2474 |  | alkylhydroperoxidase, AhpD family | 2.1 | - |  |
|  | SAV0161 | capM | capsular polysaccharide synthesis enzyme Cap5M | 2.0 | - |  |
|  | SAV2562 | crtM | squalene desaturase | 1.7 | - |  |
|  | SAV2548 | clpL | ATP-dependent Clp proteinase chain | 1.6 | - |  |
|  | SAV0152 | Cap5D | capsular polysaccharide synthesis enzyme | 1.5 | - |  |
| Metabolism | SAV2539 |  | pyruvate oxidase | 2.9 | - |  |
|  | SAV2334 |  | similar to formiminoglutamase | 2.7 | + | 2.5 |
|  | SAV2154 | glmS | D-fructose-6-phosphate amidotransferase | 2.7 | + | 2.3 |
|  | SAV0627 |  | similar to Na_ antiporter | 2.3 | - |  |
|  | SAV1875 |  | ThiJ/PfpI family protein | 2.2 | - |  |
|  | SAV0624 |  | Na+/H+ antiporter, MnhC component | 2.2 | - |  |
|  | SAV1083 | ptsH | phophocarrier protein Hpr | 2.1 | + | 7.1 |
|  | SAV2512 |  | glucarate transporter | 2.1 | - |  |
|  | SAV2309 |  | formate dehydrogenase homolog | 2.1 | - |  |
|  | SAV0628 |  | Na+/H+ antiporter, MnhG component | 2.1 | - |  |
|  | SAV2328 |  | dehydrogenase | 2.1 | - |  |
|  | SAV2581 |  | hydrolase | 2.0 | - |  |
|  | SAV0626 |  | Na_ antiporter | 2.0 | - |  |
|  | SAV2617 | nrdD | anaerobic ribonucleoside triphosphate reductase | 1.8 | - |  |
|  | SAV1881 |  | protein-tyrosine phosphatase | 1.7 | + | 2.6 |
|  | SAV0842 |  | ABC transporter ATP-binding protein homolog | 1.7 | - |  |
|  | SAV0845 | NifU | nitrogen fixation protein | 1.6 | - |  |
|  | SAV0844 |  | aminotransferase NifS homolog | 1.6 | - |  |
|  | SAV1328 |  | homoserine dehydrogenase | 1.5 | - |  |
|  | SAV1145 | trxA | thioredoxin | 1.5 | - |  |
| Protein Synthesis | SAV1224 |  | 50S ribosomal protein L28 | 2.2 | + | 2.2 |
|  | SAV0497 |  | translation initiation inhibitor homolog | 2.2 | + | 5.0 |
|  | SAV0686 | norR | transcriptional regulator | 2.0 | + | 4.8 |
| stress | SAV0840 |  | similar to sigmaB-controlled protein | 7.2 | + | 14.3 |
|  | SAV1625 | csbD | sigmaB-controlled gene product | 4.8 | + | 3.6 |
|  | SAV2182 | asp23 | alkaline shock protein 23 | 4.8 | + | 3.4 |
|  | SAV1739 |  | general stress protein-like protein | 3.6 | - |  |
|  | SAV2382 |  | similar to general stress protein | 2.7 | + | 2.5 |
|  | SAV2064 | sigB | sigma factor B | 1.5 | + | 7.2 |
|  | SAV2066 | rsbV | anti-sigmaB factor antagonist | 1.5 | - |  |
| hypothetical protein | SAV2005 |  | hypothetical protein | 7.8 | + | 5.6 |
|  | SAV2004 |  | hypothetical protein | 7.6 | + | 3.7 |
|  | SAV0374 |  | hypothetical protein | 6.9 | - |  |
|  | SAV1214 |  | hypothetical protein | 5.2 | + | 11.9 |
|  | SAV2540 |  | hypothetical protein | 4.3 | - |  |
|  | SAV2183 |  | hypothetical protein | 4.1 | + | 2.2 |
|  | SAV0823 |  | hypothetical protein | 4.0 | + | 2.1 |
|  | SAV2184 |  | hypothetical protein | 3.7 | + | 5.1 |
|  | SAV1624 |  | hypothetical protein | 3.4 | + | 10.2 |
|  | SAV0372 |  | hypothetical protein | 3.4 | - |  |
|  | SAV1082 |  | hypothetical protein | 3.3 | - |  |
|  | SAV1027 |  | hypothetical protein | 2.9 | + | 10.6 |
|  | SAV1031 |  | hypothetical protein | 2.8 | - |  |
|  | SAV1853 |  | hypothetical protein | 2.7 | - |  |
|  | SAV1854 |  | hypothetical protein | 2.6 | + | 4.1 |
|  | SAV1752 |  | hypothetical protein | 2.6 | + | 6.3 |
|  | SAV1924 |  | hypothetical protein | 2.5 | - |  |
|  | SAV1839 |  | hypothetical protein | 2.5 | + | 3.4 |
|  | SAV2085 |  | hypothetical protein | 2.5 | + | 14.8 |
|  | SAV1666 |  | hypothetical protein | 2.3 | - |  |
|  | SAV0613 |  | hypothetical protein | 2.2 | + | 7.9 |
|  | SAV0822 |  | hypothetical protein | 2.2 | - |  |
|  | SAV2342 |  | hypothetical protein | 2.0 | + | 4.2 |
|  | SAV2700 |  | hypothetical protein | 2.0 | - |  |
|  | SAV2368 |  | hypothetical protein | 2.0 | + | 3.7 |
|  | SAV2563 |  | hypothetical protein | 1.8 | - |  |
|  | SAV0571 |  | hypothetical protein | 1.8 |  |  |
|  | SAV0682 |  | hypothetical protein | 1.7 |  |  |
|  | SAV2135 |  | hypothetical protein | 1.6 |  |  |
|  | SAV1665 |  | hypothetical protein | 1.6 |  |  |
|  | SAV2647 |  | hypothetical protein | 1.5 | - |  |
|  | SAV2006 |  | hypothetical protein | 1.5 | - |  |
|  | SAV0777 |  | hypothetical protein | 1.5 | - |  |

a:+, binding to Hfq; ­-, not binding to Hfq.

b: Enrichment factor calculated by the signal intensities of Hfq IP over control IP.
