# Supplementary material for: Hfq Is a Global Regulator That Controls the Pathogenicity of Staphylococcus aureus
Source: PLoS One. 2010 Sep 29;5(9):e13069. doi: 10.1371/journal.pone.0013069 (PMC2947504; doi:10.1371/journal.pone.0013069)
Supplement: Table S3 — Genes which were enriched in the pool of Hfq IP compared to the negative control. (0.43 MB DOC) [file pone.0013069.s003.doc]

**Table S3.** Genes which were enriched in the pool of Hfq IP compared to the negative control

| MU50 ORF | MU50 gene | MU50 gene product | Enrich  ment^a^ |
| --- | --- | --- | --- |
| SAV2038 | agrC | accessory gene regulator C | 43.0 |
| SAV2227 | rpmJ | ribosomal protein L36 | 35.4 |
| SAV1650 |  | hypothetical protein | 31.4 |
| SAV1080 |  | hypothetical protein | 29.2 |
| SAV1813 | splA | serine protease | 27.0 |
| SAV1717 | EzrA | septation ring formation regulator | 26.7 |
| SAV1679 | rpmI | ribosomal protein L35 | 24.8 |
| SAV1937 | truncated-mapW | truncated map-w protein | 24.7 |
| SAV1706 |  | hypothetical protein | 21.3 |
| SAV2295 | sarR | staphylococcal accessory regulator R | 21.2 |
| SAV1811 | splC | serine protease | 21.0 |
| SAV1845 |  | hypothetical protein | 19.5 |
| SAV0320 | geh | lipase precursor | 18.6 |
| SAV2173 |  | hypothetical protein | 18.0 |
| SAV2006 |  | hypothetical protein | 17.6 |
| SAV2242 | rpmC | 50S ribosomal protein L29 | 16.8 |
| SAV2310 |  | similar to transcription antiterminator LytR | 16.5 |
| SAV1799 |  | hypothetical protein | 16.1 |
| SAV1273 | rpsO | 30Sribosomal protein S15 | 16.1 |
| SAV1800 |  | hypothetical protein | 15.6 |
| SAV1460 |  | hypothetical protein | 15.5 |
| SAV1178 |  | hypothetical protein | 15.0 |
| SAV2085 |  | hypothetical protein | 14.8 |
| SAV1045 | menB | naphthoate synthase | 14.7 |
| SAV1015 |  | hypothetical protein | 14.5 |
| SAV1405 |  | xpaC protein | 14.4 |
| SAV0840 |  | hypothetical protein | 14.3 |
| SAV2106 | atpH | ATP synthase delta chain | 14.1 |
| SAV0387 |  | hypothetical protein | 14.0 |
| SAV1335 | rpmG | ribosomal protein L33 | 13.9 |
| SAV1687 | gapB | glyceraldehyde 3-phosphate dehydrogenase | 13.0 |
| SAV1155 |  | fibrinogen binding-related protein | 12.8 |
| SAV2418 | sbi | IgG-binding protein | 12.8 |
| SAV2239 | rplX | 50S ribosomal protein L24 | 12.7 |
| SAV1343 |  | hypothetical protein | 12.4 |
| SAV0227 | pflA | pyruvate formate-lyase-activating enzyme | 12.4 |
| SAV2241 | rpsQ | 30S ribosomal protein S17 | 12.2 |
| SAV1887 |  | hypothetical protein | 12.1 |
| SAV1410 |  | hypothetical protein | 12.0 |
| SAV1421 |  | hypothetical protein | 12.0 |
| SAV1214 |  | hypothetical protein | 11.9 |
| SAV1586 | rpsT | ribosomal protein S20 | 11.9 |
| SAV1180 | ftsL | cell division protein | 11.7 |
| SAV2113 | glyA | serine hydroxymethyltransferase | 11.6 |
| SAV1646 |  | hypothetical protein | 11.6 |
| SAV0605 | adh1 | alcohol dehydrogenase | 11.3 |
| SAV1048 | sspA | V8 Protease | 11.2 |
| SAV1103 |  | hypothetical protein | 11.2 |
| SAV2121 | rho | transcription termination factor | 11.0 |
| SAV2125 | fbaA | fructose-bisphosphate aldolase | 10.9 |
| SAV2234 | rplR | ribosomal protein L18 | 10.9 |
| SAV1363 |  | 4-oxalocrotonate tautomerase | 10.9 |
| SAV1626 |  | hypothetical protein | 10.8 |
| SAV1672 | hemA | glutamyl-tRNA reductase | 10.8 |
| SAV2123 |  | hypothetical protein | 10.7 |
| SAV1567 | bex | GTP-binding protain Era homolog | 10.7 |
| SAV2236 | rpsH | ribosomal protein S8 | 10.7 |
| SAV0126 | butA | acetoin reductase | 10.7 |
| SAV1027 |  | hypothetical protein | 10.6 |
| SAV1245 | sucC | succinyl-CoA synthetase subunit beta | 10.6 |
| SAV0643 | abcA | ATP-binding cassette transporter A | 10.6 |
| SAV2238 | rplE | ribosomal protein L5 | 10.5 |
| SAV1624 |  | hypothetical protein | 10.2 |
| SAV1413 | odhA | oxoglutarate dehydrogenase | 10.2 |
| SAV1046 | sspC | cysteine protease | 10.2 |
| SAV2109 | atpB | ATP synthase subunit A | 10.1 |
| SAV0955 |  | polyribonucleotide nucleotidyltransferase | 10.1 |
| SAV1678 | rplT | 50S ribosomal protein L20 | 10.0 |
| SAV1176 |  | hypothetical protein | 10.0 |
| SAV2110 |  | hypothetical protein | 9.9 |
| SAV0232 |  | putative 3-hydroxyacyl-CoA dehydrogenase FadB | 9.8 |
| SAV2325 |  | hypothetical protein | 9.8 |
| SAV1309 | glnR | glutamine synthetase repressor | 9.8 |
| SAV1553 | sodA | superoxide dismutase | 9.7 |
| SAV0833 | gcvH | glycine cleavage system protein H | 9.5 |
| SAV2433 |  | hypothetical protein | 9.5 |
| SAV0176 |  | hypothetical protein | 9.4 |
| SAV1575 | rpsU | ribosomal protein S21 | 9.3 |
| SAV2386 | sarZ | staphylococcal accessory protein Z | 9.3 |
| SAV1506 |  | hypothetical protein | 9.2 |
| SAV1764 | rot | repressor of toxins | 9.0 |
| SAV2233 | rpsE | ribosomal protein S5 | 8.9 |
| SAV0133 |  | superoxide dismutase | 8.8 |
| SAV2252 |  | hypothetical protein | 8.8 |
| SAV0297 |  | hypothetical protein | 8.7 |
| SAV1893 |  | ferritins family protein | 8.7 |
| SAV1267 |  | hypothetical protein | 8.7 |
| SAV1228 |  | hypothetical protein | 8.7 |
| SAV2205 |  | surface protein, putative | 8.5 |
| SAV1582 | hrcA | Heat-inducible transcriptional repressor | 8.4 |
| SAV2147 |  | lytic regulatory protein truncated with Tn554 | 8.4 |
| SAV2515 |  | probable transmembrane protein smpB | 8.3 |
| SAV0934 | dltC | D-alanine--poly(phosphoribitol) ligase subunit 2 | 8.2 |
| SAV1841 | prsA | protein export protein PrsA, putative | 8.1 |
| SAV1246 | sucD | succinyl-CoA synthetase alpha subunit | 8.1 |
| SAV2554 | rocA | 1-pyrroline-5-carboxylate dehydrogenase | 8.1 |
| SAV0483 |  | hypothetical protein | 8.0 |
| SAV1107 |  | myo-inositol-1(or 4)-monophosphatase homolog | 7.9 |
| SAV1449 | recU | Holliday junction-specific endonuclease | 7.9 |
| SAV0613 |  | hypothetical protein | 7.9 |
| SAV1342 | tkt | transketolase | 7.8 |
| SAV0004 | recF | recombination protein F | 7.8 |
| SAV2120 | rpmE | ribosomal protein L31 | 7.7 |
| SAV1287 |  | hypothetical protein | 7.7 |
| SAV1189 |  | hypothetical protein | 7.7 |
| SAV2590 |  | hypothetical protein | 7.6 |
| SAV2246 | rpsS | ribosomal protein S19 | 7.6 |
| SAV2035 | hld | delta-hemolysin | 7.5 |
| SAV0568 |  | hypothetical protein | 7.5 |
| SAV0842 |  | ABC transporter ATP-binding protein homolog | 7.5 |
| SAV1564 |  | CBS domain protein | 7.3 |
| SAV1426 | dfrA | dihydrofolate reductase | 7.3 |
| SAV1063 | folD | FolD bifunctional protein | 7.3 |
| SAV0015 | rplI | 50S ribosomal protein L9 | 7.3 |
| SAV2064 | sigB | sigma factor B | 7.2 |
| SAV1736 | ccpA | catabolite control protein A | 7.2 |
| SAV1431 |  | hypothetical protein | 7.2 |
| SAV1422 |  | hypothetical protein | 7.1 |
| SAV1694 | citC | isocitrate dehydrogenase | 7.1 |
| SAV1083 | ptsH | phophocarrier protein | 7.1 |
| SAV1116 | ctaB | protoheme IX farnesyltransferase | 7.1 |
| SAV0749 |  | hypothetical protein | 7.1 |
| SAV0936 |  | nitrogen fixation protein NifU | 7.0 |
| SAV0982 |  | hypothetical protein | 7.0 |
| SAV1707 |  | putative metal-dependent hydrolase | 6.9 |
| SAV2704 | cspB | cold shock protein | 6.9 |
| SAV1306 | bsaA | glutathione peroxidase | 6.9 |
| SAV1191 | YlmH | YlmH protein | 6.8 |
| SAV2243 | rplP | 50S ribosomal protein L16 | 6.8 |
| SAV2305 |  | glycerate dehydrogenase | 6.8 |
| SAV0498 | spoVG | stage V sporulation protein G homolog | 6.8 |
| AV2226 | rpsM | 30S ribosomal protein S13 | 6.8 |
| SAV0962 | pgi | glucose-6-phosphate isomerase | 6.7 |
| SAV0537 | rplK | ribosomal protein L11 | 6.6 |
| SAV1226 |  | putative phosphatase | 6.6 |
| SAV1476 | rpsA | ribosomal protein S1 | 6.5 |
| SAV1766 |  | proline dehydrohenase homolog | 6.5 |
| SAV0251 | ispD | 2-C-methyl-D-erythritol 4-phosphate cytidylyltransferase | 6.5 |
| SAV1185 | ftsA | cell division protein | 6.5 |
| SAV1334 | katA | catalase | 6.4 |
| SAV0500 | prs | ribose-phosphate pyrophosphokinase | 6.3 |
| SAV1095 | pdhC | branched-chain alpha-keto acid dehydrogenase subunit E2 | 6.3 |
| SAV1752 |  | hypothetical protein | 6.3 |
| SAV0545 | rpsL | 30S ribosomal protein S12 | 6.2 |
| SAV1448 |  | hypothetical protein | 6.2 |
| SAV1415 | truncated-arlR | truncated (putative response regulator) | 6.1 |
| SAV1158 | efb | Fibrinogen-binding protein precursor | 6.1 |
| SAV0543 | rpoC | DNA-directed RNA polymerase beta' subunit | 6.1 |
| SAV1112 |  | hypothetical protein | 6.1 |
| SAV0957 | rocD | ornithine--oxo-acid transaminase | 6.1 |
| SAV1059 | qoxC | Quinol oxidase polypeptide III | 6.0 |
| SAV2225 | rpsK | 30S ribosomal protein S11 | 6.0 |
| SAV0535 | nusG | transcription antitermination protein | 5.9 |
| SAV1057 | fmt | fmt protein | 5.9 |
| SAV1558 |  | ATP-dependent RNA helicase | 5.9 |
| SAV1519 | recN | DNA repair protein | 5.9 |
| SAV1160 |  | hypothetical protein | 5.8 |
| AV0138 | dra | deoxyribose-phosphate aldolase | 5.8 |
| SAV0241 | lctE | L-lactate dehydrogenase | 5.8 |
| SAV1919 |  | putative manganese-dependent inorganic pyrophosphatase | 5.8 |
| SAV0110 | lctP | L-lactate permease homolog | 5.8 |
| SAV1568 | cdd | cytidine deaminase | 5.7 |
| SAV1192 |  | similar to cell-division initiation protein | 5.7 |
| SAV2005 |  | hypothetical protein | 5.6 |
| SAV1411 |  | ABC transporter homolog | 5.6 |
| SAV0333 |  | hypothetical protein | 5.5 |
| SAV1709 | ald | alanine dehydrogenase | 5.4 |
| SAV1062 |  | chitinase B | 5.4 |
| SAV1265 |  | hypothetical protein | 5.4 |
| SAV0366 | ssb | single-stranded DNA-binding protein | 5.4 |
| SAV1492 | srrA | staphylococcal respiratory response protein | 5.4 |
| SAV0002 | dnaN | DNA polymerase III subunit beta | 5.3 |
| SAV1745 |  | endo-1,4-beta-glucanase homolog | 5.2 |
| SAV1719 | rpsD | 30S ribosomal protein S4 | 5.2 |
| SAV2184 |  | hypothetical protein | 5.1 |
| SAV1209 | gmk | guanylate kinase homolo | 5.0 |
| SAV2311 |  | similar to suppressor protein suhB | 5.0 |
| SAV0497 |  | translation initiation inhibitor homolog | 5.0 |
| SAV1450 | pbp2 | penicillin-binding protein 2 | 4.9 |
| SAV0234 |  | putative acyl-CoA synthetase FadE | 4.9 |
| SAV1695 | citZ | citrate synthase | 4.9 |
| SAV1615 |  | hypothetical protein | 4.9 |
| SAV2408 |  | hypothetical protein | 4.9 |
| SAV0686 |  | transcriptional regulator | 4.8 |
| SAV2104 | atpG | ATP synthase gamma chain | 4.8 |
| SAV0730 | nrdI | ribonucleotide reductase stimulatory protein | 4.8 |
| SAV1350 | citB | aconitate hydratase | 4.8 |
| SAV0546 |  | 30S ribosomal protein S7 | 4.7 |
| SAV2145 | czrA | repressor protein | 4.6 |
| SAV0534 | secE | preprotein translocase subunit | 4.6 |
| SAV0633 |  | similar to ABC transporter ATP-binding protein | 4.5 |
| SAV1153 |  | hypothetical protein | 4.5 |
| SAV0528 |  | glutamyl-tRNA synthetase | 4.5 |
| SAV1605 |  | hypothetical protein | 4.5 |
| SAV1259 | frr | ribosome recycling factor | 4.5 |
| SAV1208 |  | fibrinogen binding protein | 4.5 |
| SAV1693 |  | hypothetical protein | 4.4 |
| AV1127 |  | hypothetical protein | 4.4 |
| SAV1675 | tig | trigger factor | 4.4 |
| SAV0235 |  | putative acetyl-CoA/acetoacetyl-CoA transferase | 4.4 |
| SAV2232 | rpmD | 50S ribosomal protein L30 | 4.3 |
| SAV2250 | rplC | 50S ribosomal protein L3 | 4.3 |
| SAV1918 |  | pyrazinamidase/nicotinamidase homolog | 4.2 |
| SAV1221 |  | hypothetical protein | 4.2 |
| SAV2342 |  | hypothetical protein | 4.2 |
| SAV1809 | splF | serine protease | 4.2 |
| SAV0386 |  | hypothetical protein | 4.2 |
| SAV1854 |  | hypothetical protein | 4.1 |
| SAV2068 |  | similar to pemK family of DNA-binding proteins | 4.1 |
| SAV0465 |  | hypothetical protein | 4.1 |
| SAV1340 |  | hypothetical protein | 4.1 |
| SAV0777 |  | hypothetical protein | 4.1 |
| SAV2171 |  | hypothetical protein | 4.0 |
| SAV1565 | glyS | glycyl-tRNA synthetase | 4.0 |
| SAV1269 | infB | translation initiation factor IF-2 | 4.0 |
| SAV0263 | lrgB | antiholin-like protein LrgB | 4.0 |
| SAV1861 |  | transcription regulator Fur family homolog | 3.9 |
| SAV2105 | atpA | ATP synthase subunit A | 3.9 |
| SAV1094 | pdhB | pyruvate dehydrogenase E1 component beta subunit | 3.9 |
| SAV1765 |  | lysophospholipase homolog | 3.8 |
| SAV0507 |  | similar to cell-division initiation protein | 3.8 |
| SAV0935 | dltD | poly (glycerophosphate chain) D-alanine transfer protein | 3.8 |
| SAV2458 |  | hypothetical protein | 3.8 |
| SAV2231 | rplO | 50S ribosomal protein L15 | 3.7 |
| SAV2135 |  | hypothetical protein | 3.7 |
| SAV0550 |  | 8-amino-7-oxononanoate synthase | 3.7 |
| SAV2004 |  | hypothetical protein | 3.7 |
| SAV0632 |  | similar to ABC transporter, permease protein | 3.7 |
| SAV0928 |  | hypothetical protein | 3.7 |
| SAV2368 |  | hypothetical protein | 3.7 |
| SAV1697 | pykA | pyruvate kinase | 3.6 |
| SAV0616 | sarA | staphylococcal accessory regulator A | 3.6 |
| SAV1310 | glnA | glutamine-ammonia ligase | 3.6 |
| SAV1625 | csbD | sigmaB-controlled gene product | 3.6 |
| SAV1885 | vraS | two-component sensor histidine kinase | 3.6 |
| SAV1738 |  | hypothetical protein | 3.6 |
| SAV2223 | rplQ | 50S ribosomal protein L17 | 3.6 |
| AV2102 | atpC | FoF1-ATP synthase epsilon subunit | 3.5 |
| SAV0566 |  | hypothetical protein | 3.5 |
| SAV1789 |  | hypothetical protein | 3.5 |
| SAV1637 | secF | protein-export membrane protein | 3.4 |
| SAV2052 |  | similar to ATP/GTP hydrolase | 3.4 |
| SAV2182 | asp23 | alkaline shock protein 23 | 3.4 |
| SAV0211 | acpD | acyl carrier protein phosphodiesterase | 3.4 |
| SAV0365 | rpsF | 30S ribosomal protein S6 | 3.4 |
| SAV0984 |  | 3-oxoacyl synthase | 3.4 |
| SAV1839 |  | hypothetical protein | 3.4 |
| SAV1268 |  | similar to ribosomal protein L7AE family | 3.4 |
| SAV1518 |  | dihydrolipoamide dehydrogenase | 3.3 |
| SAV1707 |  | putative metal-dependent hydrolase | 3.3 |
| SAV1907 |  | hypothetical protein | 3.3 |
| SAV0565 |  | hypothetical protein | 3.3 |
| SAV0647 |  | ferrichrome transport ATP-binding protein | 3.3 |
| SAV1255 | codY | transcriptional repressor CodY | 3.3 |
| SAV1557 |  | endonuclease IV | 3.3 |
| SAV2451 |  | similar to para-nitrobenzyl esterase chain A | 3.3 |
| SAV1812 | splB | serine protease | 3.3 |
| SAV1058 |  | putative quinol oxidase polypeptide IV QoxD | 3.3 |
| SAV1580 | dnaK | DnaK protein | 3.2 |
| SAV0561 | sdrC | Ser-Asp rich fibrinogen-binding, bone sialoprotein-binding protein | 3.2 |
| SAV1446 |  | hypothetical protein | 3.2 |
| SAV0284 |  | hypothetical protein | 3.2 |
| SAV2457 |  | hypothetical protein | 3.1 |
| SAV0508 |  | hypothetical protein | 3.1 |
| SAV1117 |  | hypothetical protein | 3.1 |
| SAV0221 |  | hexose phosphate transport protein | 3.1 |
| SAV1414 |  | putative protein histidine kinase | 3.1 |
| SAV1689 |  | similar to formamidopyrimidine-DNA glycosidase | 3.1 |
| SAV1515 | bmfBB | branched-chain alpha-keto acid dehydrogenase | 3.0 |
| SAV1903 |  | sex pheromone staph-cAM373 precursor | 3.0 |
| SAV2235 | rplF | 50S ribosomal protein L6 | 3.0 |
| SAV1052 |  | partial autolysin | 3.0 |
| SAV0539 |  | 50S ribosomal protein L10 | 3.0 |
| SAV1056 |  | hypothetical protein | 3.0 |
| SAV1222 | cfxE | ribulose-5-phosphate 3-epimerase homolog | 3.0 |
| SAV2164 | arg | arginase | 2.9 |
| SAV1929 |  | hypothetical protein | 2.9 |
| SAV1865 |  | hypothetical protein | 2.9 |
| SAV1562 | dnaG | DNA primase | 2.9 |
| SAV1332 |  | hypothetical protein | 2.9 |
| SAV1781 |  | transaldolase | 2.8 |
| SAV1447 |  | hypothetical protein | 2.8 |
| SAV0606 |  | hypothetical protein | 2.8 |
| SAV2646 |  | hypothetical protein | 2.8 |
| SAV1159 |  | fibrinogen-binding protein precursor | 2.7 |
| SAV1353 |  | hypothetical protein | 2.7 |
| SAV1882 |  | hypothetical protein | 2.7 |
| SAV0950 |  | hypothetical protein | 2.7 |
| SAV1207 |  | similar to PhnB protein | 2.7 |
| SAV1391 |  | hypothetical protein | 2.7 |
| SAV0981 |  | hypothetical protein | 2.7 |
| SAV1872 |  | hypothetical protein | 2.7 |
| SAV1881 |  | protein-tyrosine phosphatase | 2.6 |
| SAV1850 |  | hypothetical protein | 2.6 |
| SAV0385 |  | hypothetical protein | 2.6 |
| SAV1047 | sspB | cysteine protease precursor | 2.5 |
| SAV1184 | div1b | cell division protein | 2.5 |
| SAV0364 |  | hypothetical protein | 2.5 |
| SAV0844 |  | aminotransferase NifS homolog | 2.5 |
| SAV2382 |  | similar to general stress protein | 2.5 |
| SAV2334 |  | similar to formiminoglutamase | 2.5 |
| SAV0540 | rplL | 50S ribosomal protein L7/L12 | 2.4 |
| SAV0941 |  | putative NADH dehydrogenase | 2.4 |
| SAV2366 |  | L-lactate permease lctP homolog | 2.4 |
| SAV0538 | rplA | 50S ribosomal protein L1 | 2.4 |
| SAV2508 |  | hypothetical protein | 2.4 |
| SAV0656 |  | hypothetical protein | 2.4 |
| SAV0433 | set15 | exotoxin 15 | 2.4 |
| SAV0530 | cysS | cysteinyl-tRNA synthetas | 2.4 |
| SAV0653 |  | hypothetical protein | 2.4 |
| SAV1606 |  | acetyl-CoA carboxylase | 2.3 |
| SAV0635 |  | hypothetical protein | 2.3 |
| SAV2154 | glmS | D-fructose-6-phosphate amidotransferase | 2.3 |
| SAV1359 |  | hypothetical protein | 2.3 |
| SAV0933 | dltB | DltB membrane protein | 2.3 |
| SAV0491 |  | putative deoxyribonuclease | 2.2 |
| SAV2183 |  | hypothetical protein | 2.2 |
| SAV1224 | rpmB | 50S ribosomal protein L28 | 2.2 |
| SAV1044 |  | similar to prolyl aminopeptidase | 2.1 |
| SAV2244 | rpsC | 30S ribosomal protein S3 | 2.1 |
| SAV0160 | capL | capsular polysaccharide synthesis enzyme | 2.1 |
| SAV1404 |  | putative acylphosphatase | 2.1 |
| SAV1667 | hemL | glutamate-1-semialdehyde aminotransferase | 2.1 |
| SAV0823 |  | hypothetical protein | 2.1 |
| SAV1581 | grpE | heat shock protein | 2.1 |
| SAV2248 | rplW | 50S ribosomal protein L23 | 2.1 |
| SAV0544 |  | putative ribosomal protein L7Ae-like | 2.0 |
| SAV2228 | infA | translation initiation factor IF-1 | 2.0 |
| SAV1145 | trxA | thioredoxin | 2.0 |
| SAV0487 |  | hypothetical protein | 2.0 |
| SAV2269 | mobA | molybdopterin-guanine dinucleotide biosynthesis protein | 2.0 |
| SAV0815 | nuc | staphylococcal nuclease | 2.0 |
| SAV1073 | purH | bifunctional purine biosynthesis protein | 2.0 |

a: Enrichment factor calculated by the signal intensities of Hfq IP over control IP.
